# Transcending aging: disease specificity, assessment challenges, and management strategies for rheumatoid arthritis-related sarcopenia

**DOI:** 10.3389/fmed.2026.1890326

**Published:** 2026-08-05

**Authors:** Xuchen Ren, Shuang Ren

**Affiliations:** The First Hospital of China Medical University, Shenyang, China

**Keywords:** body composition, chronic inflammation, muscle quality, physical function, rheumatoid arthritis, sarcopenia

## Abstract

Rheumatoid arthritis (RA) is a systemic autoimmune disorder marked by chronic synovitis, with pathological damages extending beyond joint destruction to skeletal muscle, bone and metabolic homeostasis. Sarcopenia is conventionally regarded as age-driven degeneration, yet its high prevalence in young RA patients indicates a distinct disease-specific muscle phenotype mediated by persistent inflammation and joint disuse. This review establishes an integrated “muscle-bone-fat axis” framework to interpret RA-related myopathy: systemic inflammation and cumulative glucocorticoid exposure synergistically trigger myofibrillar proteolysis, enhance bone resorption and visceral fat accumulation. At the molecular level, catabolic cytokines overactivate the ubiquitin-proteasome pathway; impaired muscle-adipose crosstalk and mitochondrial dysfunction further disrupt muscle fiber integrity. We illustrate the limitations of age-based sarcopenia criteria when applied to RA populations, as active arthritis and pain distort handgrip strength assessment. We further compare muscle-related effects of diverse anti-rheumatic therapies: biological Disease-Modifying Anti-Rheumatic Drugs (bDMARDs) reverse systemic catabolism to preserve muscle mass, while JAK inhibitors exhibit superior efficacy in recovering physical performance and muscle strength. Collectively, we propose a paradigm shift from joint-centered symptomatic care to a multidisciplinary treat-to-target strategy. Early pharmacological remission combined with pain-adjusted progressive resistance training and individualized nutritional support is recommended to sustain long-term physical function in RA patients.

## 1 Introduction

Rheumatoid arthritis (RA) is a systemic autoimmune disease characterized by chronic synovitis, whose pathological impact extends beyond joints to involve muscles, bones, and metabolic function. While sarcopenia has traditionally been viewed as an age-related degenerative process, its frequent occurrence in non-elderly RA patients has prompted researchers to investigate its underlying nature. An emerging hypothesis suggests that RA-related sarcopenia might not merely represent accelerated aging, but rather could be explored as a distinct, disease-associated muscle phenotype driven by

chronic inflammation, functional limitation, and metabolic dysfunction (1). However, a comprehensive synthesis of the evidence supporting this conceptual distinction is currently lacking.

With advances in RA treatment strategies, patient survival has significantly improved. Disease-related complications, particularly abnormal body composition, have increasingly become critical factors influencing prognosis (2, 3). Sarcopenia, a syndrome characterized by reduced skeletal muscle mass, decreased muscle strength, and/or impaired physical function, is closely associated with falls, fractures, functional disability, diminished quality of life, and increased mortality risk (4, 5).

Epidemiologically, the prevalence of sarcopenia among patients with RA is significantly higher than in the general population (6), though reported rates vary widely (from ~20% to over 50%) due to the historical evolution and diverse application of diagnostic criteria (7, 8). While early definitions focused almost exclusively on isolated muscle mass reduction, modern frameworks—such as the revised European Working Group on Sarcopenia in Older People 2 (EWGSOP2) and the Asian Working Group for Sarcopenia 2019 (AWGS)—have shifted toward function-centric models prioritizing low muscle strength as the primary indicator (9, 10). However, because these consensus criteria and assessment tools were developed primarily for age-related degenerative processes in geriatric populations, concerns have been raised regarding their applicability in the RA population. These conventional approaches may not adequately account for RA-specific factors—such as the active inflammatory state, pain, and medication use—frequently leaving high-risk, hidden phenotypes like sarcopenic obesity masked behind standard body mass index (BMI) metrics (11). Consequently, the extent to which current general diagnostic criteria are applicable and accurate in RA cohorts remains a subject of ongoing clinical debate, highlighting the urgent need for a thorough evaluation of disease specificity, assessment challenges, and targeted management strategies.

The onset and trajectory of sarcopenia display distinct characteristics across the clinical phases and phenotypes of RA. Skeletal muscle depletion can initiate during the pre-clinical phase, well before clinical synovitis. In early RA, seropositive patients (rheumatoid factor, RF/anti-citrullinated protein antibodies, ACPA positive) typically experience accelerated muscle loss compared to seronegative individuals due to a higher systemic inflammatory burden (12). As the disease progresses to established RA, persistent joint destruction and chronic disuse irreversibly drive muscle fiber atrophy and myosteatosis (13).

Crucially, while aggressive anti-rheumatic therapies are pivotal for achieving clinical disease remission, control of systemic inflammation alone is often insufficient to fully restore skeletal muscle architecture or physical performance. Emerging evidence underscores that progressive resistance exercise interventions, when systematically coupled with tailored nutritional support (such as optimized protein and vitamin D supplementation), serve as indispensable, primary countermeasures. These multidisciplinary strategies directly stimulate muscle protein synthesis, counteract chronic muscle disuse, and improve long-term functional prognosis, mitigating the compounding risks of disability and falls in this fragile population (14, 15).

This narrative review aims to critically evaluate and synthesize the existing literature to address three key areas. First, we explore the evidence surrounding the hypothesized the disease-specific pathophysiological mechanisms of RA-related sarcopenia compared to age-related sarcopenia. Second, we discuss the main challenges and limitations of applying current sarcopenia assessment tools in patients with RA. Finally, we evaluate the efficacy of current intervention strategies—including anti-inflammatory, exercise, and nutritional approaches—in managing this condition.

## 2 Materials and methods

### 2.1 Literature search and selection

A comprehensive literature search was conducted in PubMed, Web of Science, and Embase databases to identify relevant studies published from database inception to March 2026.

To ensure thematic precision, explicit selection criteria were applied. Inclusion criteria comprised: (1) peer-reviewed articles published in English, including clinical trials, observational cohorts, cross-sectional studies, and systematic reviews conducted in human populations; (2) *in vivo* animal model studies (e.g., collagen-induced arthritis or adjuvant-induced arthritis models) directly examining the systemic mechanisms of inflammatory muscle mass loss and its accompanying effects on disease progression indices; (3) international consensus guidelines. Exclusion criteria comprised purely *in vitro* cell culture studies, non-RA associated muscle wasting models, and conference abstracts. Notably, regarding technical parameters, we explicitly categorized studies utilizing validated modalities for evaluating muscle mass and body composition—primarily Dual-energy X-ray Absorptiometry (DXA), Bioelectrical Impedance Analysis (BIA), Ultrasound, Magnetic Resonance Imaging (MRI), and Computed Tomography (CT)—which are critically appraised in Section 4.3.

**Figure 1:**
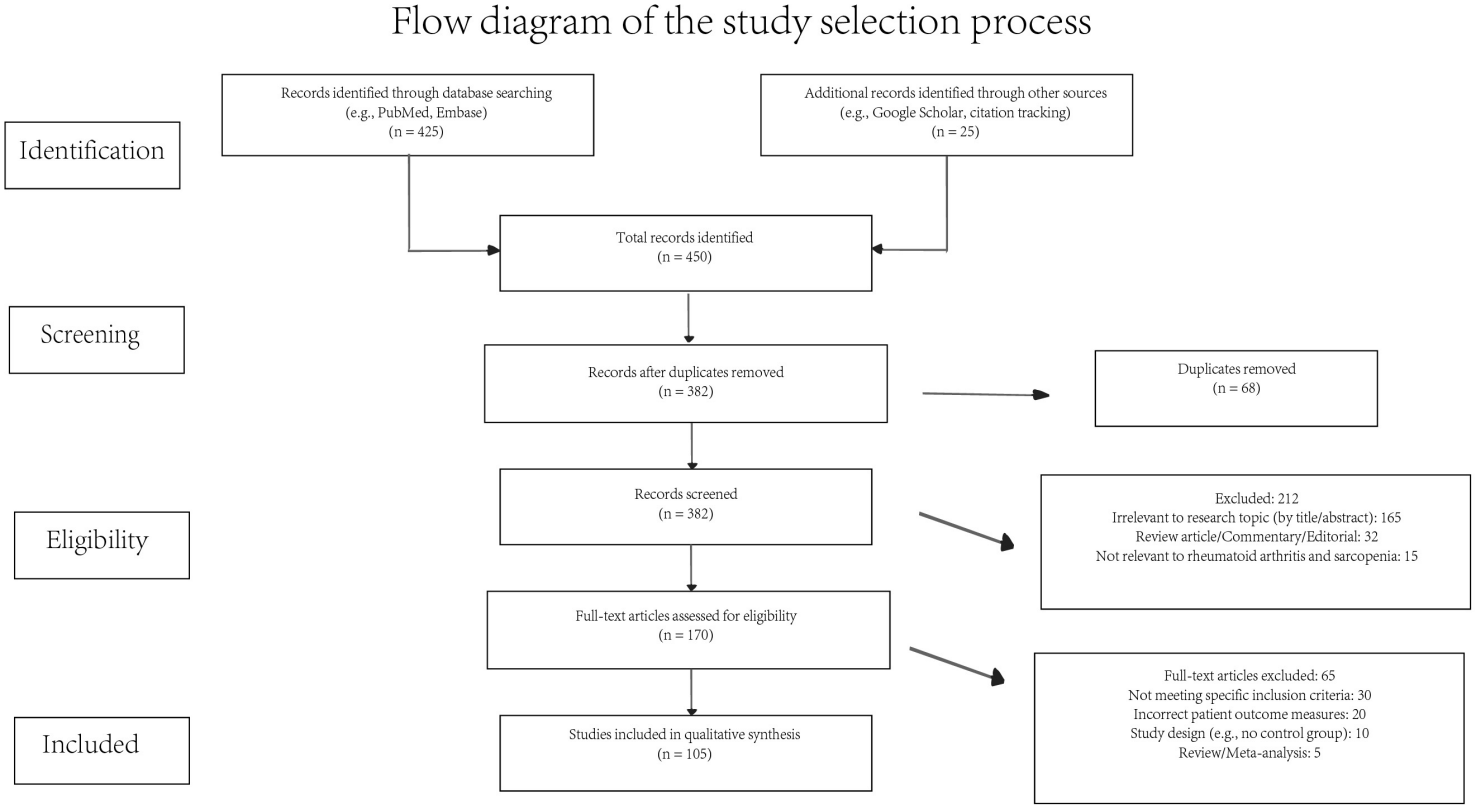
PRISMA flowchart showing the procedure of literature retrieval and screening.

Our literature search initially retrieved 450 records, and after a rigorous evaluation process (detailed in Figure 1), a final total of 105 studies were analyzed.

### 2.2 Data extraction and synthesis

Relevant data concerning study characteristics, population features, assessment methods, and key findings related to sarcopenia were extracted and synthesized qualitatively. Due to the narrative nature of this review and the substantial clinical and methodological heterogeneity across the included studies, a formal assessment of risk of bias, reporting bias, and certainty of evidence was not performed.

## 3 Concept and disease specificity of rheumatoid sarcopenia

### 3.1 Sarcopenia: from age-related to disease-related

Sarcopenia was initially regarded as a progressive degenerative change in skeletal muscle closely associated with the aging process, primarily manifested as reduced muscle mass, decreased muscle strength, and impaired physical function. Consequently, it was long classified as part of the geriatric syndrome. Early research pre-dominantly focused on physiological muscle loss due to aging, viewing it as an inevitable consequence of aging, i.e., primary sarcopenia (16). As summarized in recent narrative reviews, mounting evidence indicated that sarcopenia is not solely driven by age. It can also occur in various chronic disease states, exhibiting clinical characteristics distinct from age-related sarcopenia alone (17).

Against the backdrop of chronic inflammatory diseases, malignancies, and prolonged activity limitations, declines in muscle mass and function often occur pre-maturely. The underlying mechanisms are closely associated with persistent inflammation, metabolic disorders, malnutrition, and reduced physical activity. This type of sarcopenia is typically classified as secondary or disease-related sarcopenia. Accumulating evidence indicates that, compared to age-related sarcopenia, disease-related sarcopenia can occur in non-elderly populations, may progress more rapidly, and is closely associated with the activity status and treatment of the underlying disease (18, 19).

In recent years, multiple international consensus statements have increasingly recognized that viewing sarcopenia solely as an age-related disease fails to fully capture its clinical essence (20). The prevailing view now emphasizes shifting from a traditional “age-centric” perspective to a comprehensive “disease- and pathology-oriented” approach to more accurately identify the risk of sarcopenia and its potential reversibility across different populations. This conceptual shift provides the theoretical foundation for exploring the mechanisms, assessment methods, and intervention strategies of sarcopenia within specific disease contexts, such as RA.

Crucially, within this multi-factorial framework, chronological age and sex operate as decisive biological variables that distinctively modulate the development and phenotypic expression of sarcopenia in RA. Evidence across stratified clinical cohorts indicates that the impact of age is non-linear and phenotype-specific. In younger and middle-aged RA populations (under 50 years), skeletal muscle depletion is primarily driven by high disease activity and excessive systemic inflammatory catabolism, often presenting pre-maturely. Conversely, in elderly RA patients (aged 50 years and above), the chronic inflammatory pathology of RA directly synergizes with the physiological signaling of age-related primary sarcopenia and cellular senescence, leading to an accelerated, compounding trajectory of muscle wasting and functional impairment (21).

Furthermore, sex-specific dimorphism significantly dictates these body composition trajectories. Female RA patients, particularly postmenopausal women, exhibit a markedly higher susceptibility to both sarcopenia and the “sarcopenic with hyperadiposis” (sarcopenic obesity) phenotype compared to age-matched males (22, 23). This female pre-disposition is highly correlated with the loss of estrogenic protective effects on muscle protein turnover, an inherent tendency for visceral adiposity accumulation under chronic inflammation, and gender-based differences in baseline specific muscle strength. Therefore, a comprehensive understanding of rheumatoid sarcopenia requires careful clinical stratification based on both age ranges and sex-specific risk profiles.

### 3.2 Body composition changes in patients with RA

Patients with RA commonly exhibit significant body composition abnormalities, characterized by reduced muscle mass and lean body mass, increased fat mass, and abnormal fat distribution. A recent narrative synthesis of multiple studies indicates that compared to healthy controls, RA patients have significantly lower lean body mass and significantly higher fat indices, even when BMI is similar (24). Real-world cohort studies indicate that the prevalence of sarcopenia and reduced muscle function in RA patients reaches approximately 36. 5% (21). Additionally, patients with early RA exhibit higher body fat percentages and lower muscle mass, with these adverse body composition features correlating with inflammatory markers (25). Specific cohort studies further confirm that reduced muscle mass and increased fat mass are particularly pronounced in female RA patients, accompanied by a higher prevalence of sarcopenic obesity (26).

Regarding skeletal muscle, RA patients frequently exhibit abnormal body composition, with lean body mass and limb muscle mass significantly lower than age- and sex-matched healthy controls. Further studies reveal that compared to general reference populations, RA patients have markedly higher prevalence of low lean body mass, obesity, and sarcopenic obesity, closely associated with functional decline (27). More recent studies indicate that approximately half of RA patients exhibit sarcopenic status, with the majority concurrently presenting a sarcopenic obesity phenotype. This suggests that abnormal body composition is highly prevalent in RA and impacts clinical outcomes (22).

An association has been demonstrated between chronic inflammatory phenotypes and sarcopenia development, implying that muscle loss in RA may be mediated by disease-related inflammation rather than solely age-related degenerative changes (28). This systemic microenvironment drives a destructive muscle–fat crosstalk. In the skeletal muscle niche, overexpression of the catabolic myokine myostatin directly represses muscle hypertrophy, while protective irisin is suppressed. Concurrently, elevated chemokines, including CCL2 and CX3CL1, recruit pro-inflammatory M1 macrophages into the muscle interstitium. This cascade hyperactivates the Ubiquitin-Proteasome System (UPS) and upregulates E3 ligases (MuRF1 and Atrogin-1), while impairing satellite cell regeneration, collectively reducing muscle mass independently of aging (29).

Altered adipose tissue constitutes another critical component of RA's abnormal body composition. RA patients exhibit increased fat mass and abnormal fat distribution, particularly marked by a significant rise in visceral adipose tissue (VAT) proportion—a change independent of conventional BMI metrics (i.e., normal BMI with elevated VAT) (30). Compared to healthy controls, RA patients exhibit a general trend of reduced muscle mass and increased fat mass (31). This abnormal body composition manifests not only as excess fat but also as muscle loss, forming so-called sarcopenic obesity. Its prevalence is markedly higher in RA than in the general population and correlates with functional decline and reduced quality of life (24, 32). As a highly metabolically active and endocrine-secreting tissue, visceral fat releases multiple pro-inflammatory cytokines. These adipokines are implicated in amplifying inflammation and metabolic abnormalities, thereby exacerbating the systemic inflammatory state in RA. Consequently, relying solely on weight or BMI fails to accurately reflect the true nutritional and functional status of RA patients.

Consequently, relying solely on universal BMI thresholds fails to accurately reflect the true metabolic and clinical status of RA patients due to significant cutoff variability and the masking effect of severe fat infiltration into muscle tissue (myosteatosis). To overcome this, specific modalities are utilized, each with diagnostic trade-offs. DXA is the reproducible clinical gold standard for quantifying lean mass, though BIA offers a faster, non-invasive alternative despite being easily confounded by joint edema. For advanced tissue quantification, MRI and CT represent definitive research gold standards for tracking myosteatosis, but high costs limit their routine use. Alternatively, bedside Ultrasound is emerging as an accessible tool to monitor muscle thickness and quality via echointensity (33).

RA patients frequently experience reduced bone mass or even osteoporosis, which, together with skeletal muscle loss and abnormal fat distribution, constitutes the so-called “muscle-bone-fat axis” imbalance. Chronic inflammation, prolonged glucocorticoid use, reduced physical activity, and endocrine disorders are all considered key drivers of this imbalance. Multidimensional alterations in body composition not only increase the risk of falls, fractures, and functional impairment in RA patients but also are closely associated with a higher risk of cardiovascular events and all-cause mortality.

**Figure 2:**
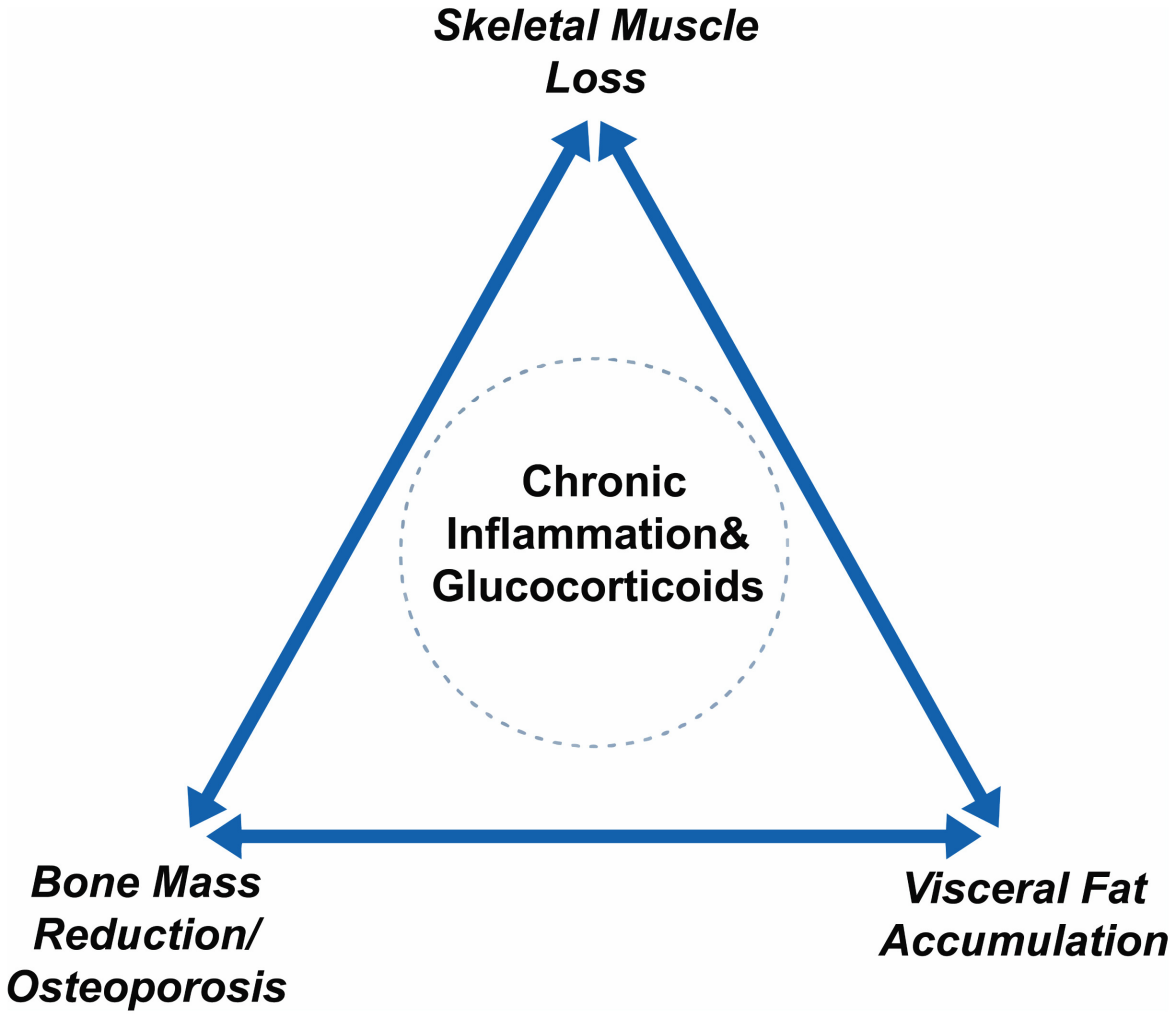
Schematic diagram of the muscle–bone–fat axis in RA. Systemic chronic inflammation and cumulative glucocorticoid exposure serve as the central upstream drivers that disrupt the homeostasis of this tripartite axis. Elevated catabolic cytokines synergistically accelerate myofibrillar proteolysis to cause muscle loss, while promoting visceral fat accumulation and myosteatosis. Simultaneously, this inflammatory microenvironment stimulates osteoclastogenesis and suppresses osteoblastogenesis, leading to progressive bone mass reduction and osteoporosis. The reciprocal feed-forward loops among muscle wasting, bone loss, and fat gain collectively compromise joint stability, compounding the risk of falls and physical disability.

Importantly, body composition trajectories differ across disease stages. While sarcopenic obesity can emerge in early RA, absolute muscle mass loss and severe functional decline are significantly more pronounced in established RA due to the cumulative burden of joint damage and prolonged inactivity. The interactions among skeletal muscle, bone, and VAT in RA can be summarized as a muscle–bone–fat axis, as illustrated in Figure 2.

### 3.3 Rheumatoid cachexia and sarcopenia: overlap and distinctions

Rheumatoid cachexia (RC) and sarcopenia are both common types of body composition abnormalities in RA patients. While there is some overlap in their clinical manifestations, their conceptual definitions and primary focuses differ.

RC is considered a relatively RA-specific body composition alteration, primarily characterized by a lack of significant fat mass reduction or even fat mass increase, coupled with lean body mass loss. It can manifest in the early stages of RA and is not strongly driven by aging (34). Under chronic inflammation, dysregulation among skeletal muscle satellite cells, macrophages, and fibroblasts forms a key pathological basis, suggesting RC is an inflammation-driven complex process rather than simple age-related degeneration (35). As highlighted in recent narrative reviews, due to inconsistent diagnostic criteria, reported RC prevalence varies widely across studies (approximately 1%−67%) (14). Concurrently, abnormal body composition in RA patients is closely associated with metabolic status and pro-inflammatory adipokine levels, providing evidence for understanding the inflammation-metabolism interaction mechanisms underlying RC (36). Sarcopenia is a muscle disorder characterized by a reduction in skeletal muscle mass, accompanied by decreased muscle strength and/or impaired physical function. Although originally regarded as an age-related condition, recent international consensus has extended its definition to multiple chronic diseases, including RA. In RA patients, the onset of sarcopenia is additionally affected by disease activity, inflammatory burden, reduced physical activity, and clinical treatment regimens (37).

**Figure 3:**
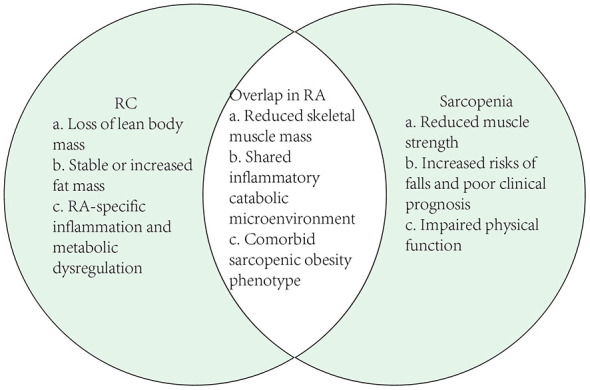
Venn diagram illustrating distinct characteristics and overlapping phenotypes between RC and sarcopenia in RA patients. RC fundamentally represents an inflammation-driven metabolic reprogramming, primarily characterized by muscle mass depletion with stable or paradoxically increased adiposity. Sarcopenia, in contrast, is a function-centric muscle disorder that prioritizes the progressive loss of muscle strength and physical performance, directly escalating the risk of falls and joint instability. The overlapping region highlights their shared catabolic microenvironment, wherein persistent systemic inflammation and joint deconditioning synergize to drive muscle atrophy and facilitate the development of the “sarcopenic obesity” phenotype.

In RA patients, both conditions often coexist. Patients with RC frequently exhibit reduced muscle mass, with some further developing muscle weakness or functional impairment that meets the diagnostic criteria for sarcopenia. They may also present with phenotypes such as sarcopenic obesity (21). However, their emphases differ: RC emphasizes inflammation-related changes in body composition and metabolism, while sarcopenia focuses on impaired muscle function and its clinical consequences, such as its closer association with reduced mobility and joint damage markers (35). Furthermore, sarcopenia does not necessarily present with a typical RC phenotype. The sequence of onset, driving mechanisms, and functional impacts may vary between the two conditions in different RA patients, as highlighted in recent narrative reviews on RA sarcopenia (7). In summary, a Venn diagram is provided to visually demonstrate the phenotypic distinctions and overlapping pathogenic features between RC and sarcopenia, as detailed in Figure 3.

### 3.4 Key differences between RA-related sarcopenia and age-related sarcopenia

Although both RA-associated sarcopenia and age-related sarcopenia phenotypically manifest as reduced skeletal muscle mass, potentially accompanied by diminished muscle strength and physical function, they exhibit significant differences in their underlying context, pathophysiological mechanisms, and clinical characteristics (as shown in Table 1).

**Table 1:** Key differences between age-related sarcopenia and RA-related sarcopenia.

Feature	Age-related sarcopenia (40, 41)	RA-related sarcopenia (42)	Clinical take-home message
Primary driver	Aging, hormonal decline, physical inactivity	Chronic inflammation, joint damage, drug toxicity	Dual-target therapy: anti-inflammatory control is prerequisite for resistance exercise
Onset and trajectory	Gradual, progressive decline over decades	Early onset, rapid deterioration during flares	Initiate sarcopenia screening upon RA diagnosis independent of age
Body composition	General muscle atrophy, often accompanied by overall weight loss	Prevalent sarcopenic obesity; muscle loss masked by fat gain	BMI cannot reflect hidden muscle depletion in RA patients
Muscle quality	Slow decline in fiber size and contractility	Rapid myosteatosis and early-onset reduced specific strength	Evaluate HGS first, as weakness precedes muscle-mass loss

BMI, body mass index; RA, rheumatoid arthritis; HGS, hand grip strength.

Regarding affected populations and temporal progression, age-related sarcopenia primarily occurs in the elderly and represents a gradually advancing physiological-pathological continuum with aging. In contrast, RA-Related Sarcopenia can develop in non-elderly RA patients and may even emerge in the early stages of the disease. For instance, a prospective study of newly diagnosed RA patients found that approximately 31.5% exhibited a sarcopenic phenotype at initial diagnosis. Notably, the average age of these patients was not restricted to the elderly, suggesting that pathological factors inherent to RA exert an independent influence on muscle loss (38). Furthermore, comprehensive body composition studies demonstrate reduced lean body mass in RA patients even within younger subgroups, showing no direct correlation with age (26). Additionally, nationwide cohort analyses support the notion that sarcopenia in RA correlates with disease pathology rather than simply increasing with age (39).

Pathologically, age-related sarcopenia primarily involves hormone level changes associated with aging, neuromuscular functional decline, mitochondrial dysfunction, and reduced physical activity. In contrast, as detailed in recent narrative reviews. RA-related sarcopenia more prominently reflects the impact of chronic systemic inflammation. In RA patients, persistent inflammatory burden and pro-inflammatory cytokines [e.g., tumor necrosis factor-α (TNF-α), interleukin-6 (IL-6), interleukin-1 (IL-1)] accelerate skeletal muscle loss and functional decline by enhancing protein breakdown, suppressing muscle synthesis, and disrupting muscle regeneration processes (1). Clinical data indicate significantly elevated inflammatory factors (IL-1α, IL-6, TNF-β) in RA-related sarcopenia patients, further supporting inflammation-mediated muscle loss mechanisms (28). As summarized in recent narrative reviews of pre-clinical animal models, inflammation, activation of protein degradation pathways, and interference with synthesis and regeneration collectively contribute to RA-related sarcopenia (43). Furthermore, reduced physical activity due to chronic pain, limited mobility, and joint damage exacerbates muscle metabolic imbalance—a point emphasized in narrative reviews of RA-related sarcopenia (7).

Regarding body composition characteristics, age-related sarcopenia typically manifests as isolated declines in muscle mass and strength, whereas RA-Related Sarcopenia is more frequently accompanied by increased fat mass or abnormal fat distribution, presenting a “muscle loss with excess fat” profile. For example, DXA studies reveal that compared to healthy controls, RA patients exhibit significantly reduced lean body mass and markedly increased fat mass, particularly higher fat percentage, a change masked even when BMI remains within the normal range (26). Furthermore, studies report a high prevalence of sarcopenic obesity among RA patients with sarcopenia, further suggesting that excess fat is a common manifestation of RA-Related Sarcopenia (22). Research on the correlation between adipokines and abnormal body composition also indicates that RA patients with higher fat mass are more prone to low lean body mass, suggesting that inflammation and fat metabolism jointly contribute to the process of body composition alteration (44).

Regarding clinical significance and assessment priorities, age-related sarcopenia primarily evaluates fall, disability, and mortality risks in the elderly. In contrast, RA-Related Sarcopenia is closely linked not only to these outcomes but also to disease activity, functional impairment, reduced quality of life, and long-term prognosis. Therefore, applying the age-related sarcopenia assessment framework alone to RA patients may underestimate its true prevalence and clinical impact.

In summary, RA-Related Sarcopenia is hypothesized to be more than merely an accelerated aging process, potentially manifesting as a disease-specific muscle phenotype characterized by distinct mechanisms and body composition features emerging within a chronic inflammatory context. Clarifying its distinctions from age-related sarcopenia facilitates optimized risk assessment and individualized management strategies for RA patients.

## 4 Challenges in assessing and diagnosing RA-associated sarcopenia

### 4.1 Overview of current sarcopenia diagnostic criteria

Diagnostic criteria for sarcopenia have evolved from a sole focus on muscle mass to a comprehensive assessment of muscle function and body composition. In recent years, multiple international academic organizations have successively proposed and updated diagnostic frameworks for sarcopenia, aiming to enhance the accuracy of clinical identification and the comparability of research results.

The EWGSOP2 consensus, updated in 2019, is widely applied in clinical and research settings. This consensus explicitly identifies reduced muscle strength as the core characteristic of sarcopenia, establishing “low muscle strength” as the primary criterion for screening and diagnosis. Confirmation of sarcopenia requires this to be combined with low muscle mass, with disease severity graded using physical function indicators. This function-oriented diagnostic approach significantly enhances sarcopenia's predictive value for adverse clinical outcomes (9).

Addressing differences in body composition and physique among Asian populations, the AWGS released and updated its sarcopenia diagnostic consensus in 2019. This standard retains the three-tiered assessment framework of “muscle strength—muscle mass—physical function” while proposing specific thresholds tailored for Asian individuals (45). Further evidence-based research indicates that sarcopenia diagnosis based on the AWGS 2019 criteria correlates with relevant health outcomes, and the introduction of the “probable sarcopenia” classification aids in early identification of high-risk individuals in community populations (46). Additionally, compared to traditional criteria, the AWGS 2019 update significantly increased the detection rate of severe sarcopenia among hospitalized patients, suggesting improved applicability and sensitivity across different clinical settings (47). Related meta-analyses also indicate a growing number of studies on sarcopenia prevalence in Asian populations using the AWGS 2019 criteria, further demonstrating the broad application potential of these standards in epidemiological and clinical populations (48).

In recent years, the Global Leadership Initiative in Sarcopenia (GLIS) has further unified the conceptual definition of sarcopenia, explicitly stating it as a muscle disorder occurring across multiple chronic disease states rather than solely an aging syndrome. This perspective provides a crucial theoretical foundation for sarcopenia research and clinical application in non-geriatric chronic inflammatory diseases like RA (4).

Despite global population variability, the revised EWGSOP2 consensus is widely considered the best operational benchmark worldwide, a position further consolidated by GLIS. Its primary advantage in clinical practice is the shift from mass-centric to function-centric models, establishing low muscle strength as the primary diagnostic indicator to drastically enhance early screening sensitivity within busy outpatient workflows.

When applied to RA cohorts, these diagnostic criteria exhibit marked variability in diagnostic sensitivity and clinical relevance. The revised EWGSOP2 framework demonstrates high sensitivity in identifying probable sarcopenia in active RA due to its heavy prioritization of muscle strength, though its primary advantage—simplicity—can be confounded by localized joint flares (4, 49). Conversely, the AWGS 2019 consensus provides higher phenotypic specificity for Asian RA populations by utilizing region-specific cut-offs (50). Cross-sectional evidence indicates that under these commonly used criteria, higher sarcopenia detection rates are tightly associated with clinical factors including advanced age, prolonged disease duration, elevated disease activity indices (DAS28-CRP: disease activity score in 28 joints with C-reactive protein), and worsening radiographic joint damage markers (22).

In our view, while international criteria converge on a tripartite diagnostic model, these phenotypes carry asymmetrical clinical weight in the context of chronic autoimmunity. We argue that low muscle strength (handgrip strength, HGS; 5-chair stand test, 5-CST) serves as a hyper-sensitive but highly confounded screening marker in RA, where active peripheral joint destruction and kinesiophobia frequently trigger functional false-positives. Consequently, we contend that structural muscle quantity reduction remains the only unambiguous diagnostic anchor for confirmed rheumatoid sarcopenia. Lastly, the impaired physical performance phenotype (gait speed) should be critically interpreted not merely as severe sarcopenia, but as a longitudinal cumulative marker reflecting the synergistic erosion of systemic cachexia, neurological fatigue, and joint deformities.

### 4.2 Limitations of existing standards in RA populations

Although current international consensus provides a relatively unified diagnostic framework for sarcopenia, these standards were primarily established based on elderly or general populations, and their applicability in RA populations remains significantly limited.

First, the diagnostic pathway centered on reduced muscle strength is susceptible to interference from joint pathology. RA patients often experience diminished grip strength or lower limb power due to joint pain, swelling, and structural damage. Such functional limitations do not fully reflect primary muscle dysfunction, potentially leading to overestimation of sarcopenia and reduced diagnostic specificity (21).

Second, RA-specific body composition abnormalities weaken the diagnostic accuracy of muscle mass-based indicators. RA patients frequently exhibit concurrent reductions in muscle mass and increases in fat mass, presenting a “sarcopenic with hyperadiposis” body composition pattern. This maintains body weight or BMI at normal or even elevated levels, masking underlying low lean body mass status. Furthermore, review evidence indicates that reduced muscle mass and increased fat mass in RA patients are observable across different genders and age groups, with this trend emerging even in early RA (24).

Furthermore, the disease applicability of current diagnostic thresholds remains inadequately validated. Even within the same diagnostic framework, RA sarcopenia prevalence rates vary significantly across studies, suggesting that directly applying general population thresholds may fail to accurately reflect disease characteristics in RA patients (1). A critical appraisal reveals significant diagnostic variability: applying EWGSOP2 vs. AWGS 2019 criteria to the same RA cohorts yields discordant prevalence rates. This heterogeneity complicates cross-study comparisons and underscores the need for RA-specific thresholds.

In summary, the application of existing sarcopenia diagnostic criteria in the RA population is constrained by multiple factors. It is necessary to incorporate RA-specific considerations within the existing consensus framework to enhance diagnostic accuracy and clinical utility.

### 4.3 Assessment of muscle mass and muscle quality

In sarcopenia assessment, muscle mass and muscle quality represent distinct dimensions of muscle health. As shown in Table 2, various measurement methods enable multidimensional evaluation of muscle mass and quality. Traditional assessments often emphasize reduced muscle mass; however, in chronic inflammatory diseases like RA, alterations in muscle structure and function frequently precede or occur independently of mass loss. Relying solely on muscle mass indicators may underestimate actual muscle impairment (13).

**Table 2:** Diagnostic steps, recommended tools and clinical guidance for sarcopenia assessment.

Diagnostic step	Recommended tools	Clinical take-home/prioritization guidance
SCREEN (identify at-risk patients)	SARC-F Questionnaire (54), Clinical suspicion (fatigue, falls, active RA)	First-line screening: zero-cost, rapid; routine use in rheumatology outpatient clinics
ASSESS (evaluate muscle strength/quality)	HGS, 5-CST (55)	Mandatory early step: strength declines before mass. It is the most reliable early indicator of poor muscle quality in RA (1)
CONFIRM (quantify muscle mass)	DXA (Gold standard for clinical use), BIA (Alternative) (56), US (myosteatosis evaluation)	Targeted detection: only applied after abnormal screening/strength results. Bedside ultrasound enables radiation-free evaluation of muscle fat infiltration (57)
SEVERITY (assess physical performance)	Gait speed, TUG, SPPB (58, 59)	Prognostic evaluation: reserved for confirmed sarcopenia, to stratify severity; predicts late-RA disability, fall risk and mortality

SARC-F, sarcopenia-frequency questionnaire; US, ultrasound; TUG, timed up and go test; SPPB, short physical performance battery; 5-CST, 5-chair stand test; BIA, bioelectrical impedance analysis; DXA, dual-energy X-ray absorptiometry; HGS, hand grip strength; RA, rheumatoid arthritis.

RA patients commonly exhibit inflammation-related muscle metabolic abnormalities. Even when overall muscle mass changes are minimal, increased intramuscular fat deposition, altered muscle fiber composition, and reduced contraction efficiency may occur. Collectively termed diminished muscle quality, these alterations are closely associated with muscle weakness and impaired physical function, and to some extent independent of body weight or BMI (51, 52).

Therefore, in the RA population, sarcopenia assessment should extend beyond single muscle mass indicators and incorporate comprehensive evaluation strategies reflecting muscle structure and functional characteristics to more accurately characterize disease-related muscle abnormalities and enhance clinical interpretability.

Currently, universally established clinical cut-offs for muscle quality in RA (e.g., specific CT attenuation or ultrasound echo intensity thresholds) are lacking. For practical clinical interpretation, we recommend a tiered approach: while MRI and CT serve as research gold standards, ultrasound (echo intensity) is the most accessible tool for routine clinics (53). If imaging is unavailable, “specific strength” (e.g., HGS/lean mass) offers a reliable proxy for muscle quality (1). Clinically, muscle quality evaluation must be integrated into diagnostic algorithms, especially for RA patients with unexplained functional decline but “normal” muscle mass, to prevent underdiagnosis.

### 4.4 Functional assessment and RA-specific considerations

Functional indicators (e.g., muscle strength, walking speed, and standing ability) are crucial components for predicting adverse outcomes in sarcopenia assessment. However, interpreting functional assessments in RA populations requires careful consideration of disease-specific factors.

Functional limitations in RA patients often stem not only from muscle dysfunction but are significantly influenced by joint pain, swelling, deformity, and restricted mobility. Studies indicate that grip strength and lower limb function test results in RA patients correlate closely with disease activity and pain levels, rather than fully reflecting actual muscle contraction capacity. This discrepancy may lead to an overestimation of sarcopenia risk (60).

Furthermore, the level of inflammatory activity and treatment status in RA exert dynamic effects on functional performance. Active inflammation exacerbates functional impairment through pain and fatigue, while some functional indicators may improve with inflammation control and disease remission. This fluctuating nature makes it challenging for single-time-point functional assessments to comprehensively reflect the long-term muscle health status of RA patients. Cohort studies demonstrate significant correlations between RA disease activity and functional scores (e.g., GLFS-25: geriatric locomotive function scale), with inflammatory fluctuations over time predicting changes in long-term functional status (61).

Therefore, when interpreting sarcopenia-related functional indicators in the RA population, it is essential to integrate disease activity, pain levels, and joint function status for a comprehensive assessment. Combining functional evaluation with measures of muscle mass and muscle quality enhances the accuracy and clinical utility of sarcopenia assessment in RA patients.

### 4.5 Future directions in diagnostics

As understanding of RA-related sarcopenia deepens, future diagnostic approaches will progressively transcend the limitations of traditional muscle mass and single functional indicators, evolving toward more comprehensive, dynamic, and individualized assessments.

First, multidimensional assessment frameworks are emerging as a key trend in sarcopenia diagnosis. Compared to single indicators, integrated strategies combining muscle mass, muscle quality (e.g., intramuscular fat infiltration, muscle density), and muscle function—alongside relevant biomarkers—hold promise for improving the sensitivity and specificity of sarcopenia identification in RA populations. Existing research indicates that quantitative imaging-based assessment methods (such as MRI or ultrasound measurements of muscle cross-sectional area, echogenicity, and fat infiltration) correlate closely with muscle strength and physical function status. These methods can serve as important supplements to conventional assessment systems, contributing to a more comprehensive reflection of muscle health in RA patients (52, 62).

Furthermore, to address the limitations of single-time-point assessments, dynamic monitoring and digital technologies are emerging as key directions for long-term sarcopenia management. Wearable devices, mobile health applications, and remote assessment platforms can capture real-time data on daily activity levels, exercise behaviors, and functional changes, providing continuous functional variable data. This offers more comprehensive information support for individualized assessment and clinical intervention. For example, digital health technologies based on sensors in smartphones and smartwatches can remotely capture activity and functional metrics in RA patients. Combined with patient-reported outcomes, this improves characterization of daily functional status and understanding of disease impact (63).

In summary, future sarcopenia diagnostics will achieve integrated optimization in technological approaches, assessment dimensions, and personalized strategies, enhancing the early identification and precise management of RA-related sarcopenia through a more comprehensive and dynamic perspective.

## 5 Pathophysiological mechanisms: clinical overview

**Figure 4:**
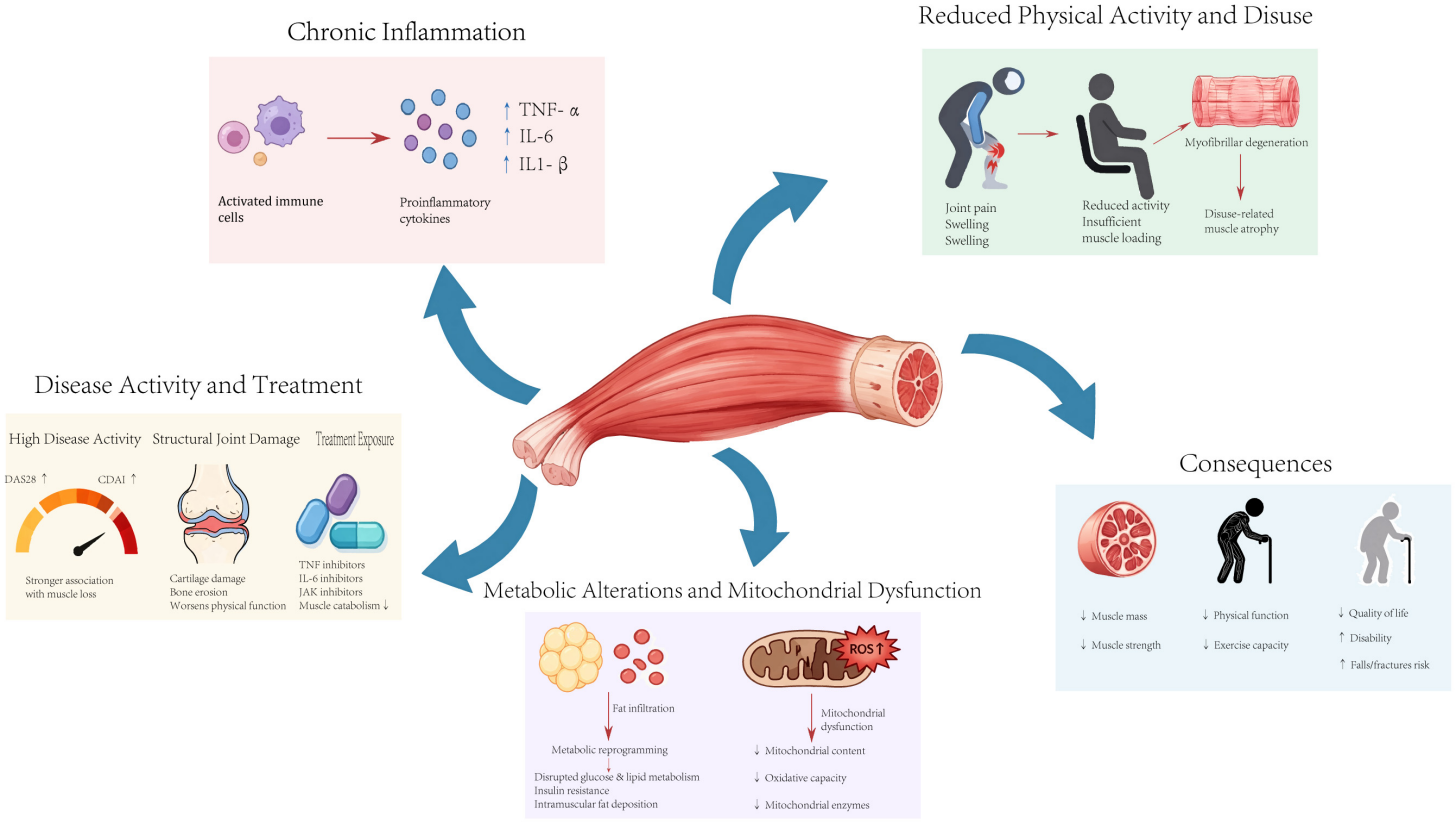
Schematic diagram of the core pathophysiological mechanisms driving muscle loss in RA-related sarcopenia. The pathogenesis of RA-related sarcopenia is defined by a multi-factorial network comprising systemic chronic inflammation, disuse, and metabolic dysregulation. Circulating pro-inflammatory cytokines (such as TNF-α and IL-6) accelerate myofibrillar protein breakdown via the UPS while suppressing muscle anabolic signaling. Joint pain and physical deconditioning lead to a disuse-related cascade that drives muscle atrophy, which can be reversed through PRT. Simultaneously, mitochondrial dysfunction and intracellular ROS accumulation cause a state of bioenergetic deficit and metabolic reprogramming, compounding muscle mass decline. Additionally, high disease activity and structural joint damage directly worsen physical function, while targeted therapies (such as bDMARDs and JAK inhibitors) mitigate muscle catabolism by suppressing systemic inflammation. These interacting clinical exposures and pathophysiological pathways ultimately culminate in a synchronized depletion of muscle mass, strength, and physical performance.

RA-associated sarcopenia is multifactorial, driven by cumulative RA-specific pathogenic factors over time. Chronic systemic inflammatory response, disuse-induced hypoactivity, disturbed intramuscular metabolism and mitochondrial dysfunction, alongside high disease activity and DMARD treatment effects, jointly impair skeletal muscle integrity and function, which serves as the central pathological framework underlying RA-related sarcopenia. Figure 4 schematically summarizes these four interconnected upstream mechanisms that cooperate to induce muscle loss and related adverse clinical outcomes in RA.

### 5.1 Chronic inflammation and muscle catabolism

Chronic systemic inflammation represents one of the most central drivers of RA-Related Sarcopenia. Persistently elevated proinflammatory cytokines (e.g., TNF-α, IL-6, IL-1β) in RA patients directly activate muscle protein catabolic pathways while suppressing anabolic signaling, leading to net muscle loss. Research indicates that this inflammation-mediated catabolic state is closely associated with reduced muscle mass, decreased muscle strength, and impaired physical function, existing to some extent independently of age factors (1). Furthermore, proinflammatory cytokines are significantly elevated in RA patients with sarcopenia, supporting the link between inflammation itself and muscle loss mechanisms (28). Persistent inflammation may further exacerbate muscle mass deterioration by disrupting the muscle microenvironment and impairing satellite cell regenerative capacity, making RA patients more susceptible to progressive muscle dysfunction (35).

Specifically, elevated circulating TNF-α and IL-6 orchestrate the pathological activation of intracellular janus kinase-signal transducer and activator of transcription (JAK-STAT) and p38 MAPK signaling pathways within skeletal muscle fibers (64). This cascade upregulates key muscle-specific E3 ubiquitin ligases—specifically muscle RING finger 1 (MuRF1) and muscle atrophy F-box (MAFbx/Atrogin-1)—thereby hyperactivating the UPS to accelerate the proteolysis of myofibrillar contractile proteins. A comprehensive original research article further elaborated the downstream molecular basis of MuRF1-mediated protein breakdown, identifying UBE2L3 as the essential E2 conjugating enzyme partner of MuRF1/TRIM63 that directly participates in the ubiquitination and degradation of myofibrillar actin and myosin (65). Concurrently, these pro-inflammatory pathways suppress the anabolic Akt/mTOR signaling axis, effectively arresting protein translation and muscle hypertrophic remodeling (66).

Additionally, the mechanistic landscape extends beyond classic cytokines to include altered muscle-fat crosstalk; systemic inflammation disrupts the balance of key myokines (e.g., elevated myostatin, decreased irisin) and adipokines (e.g., leptin, adiponectin), ultimately driving secondary metabolic dysfunctions like insulin resistance that further accelerate muscle catabolism (1).

### 5.2 Impact of reduced physical activity and muscle disuse

Reduced physical activity and disuse-related muscle atrophy are significant amplifying factors in RA-Related Sarcopenia. RA patients often experience markedly reduced daily activity due to joint pain, swelling, fatigue, and functional limitations. This prolonged state of low activity exacerbates insufficient muscle loading and myofibrillar degeneration, further driving declines in muscle mass and strength. A recent observational, cross-sectional study show that physical activity levels in RA patients are generally lower than in healthy controls. Inadequate activity correlates with higher pain scores, greater fatigue, and poorer outcomes on functional questionnaires, suggesting reduced activity may contribute to sarcopenia progression (67). Furthermore, clinical cohort studies indicate that reduced muscle mass is highly correlated with impaired physical function in early-stage RA patients. Those with higher disease activity exhibit more severe muscle loss and functional impairment, supporting the “reduced activity-disuse-sarcopenia” association (12).

Mechanistically, tailored physical activity dampens active RA by interrupting systemic TNF-α/IL-6 pathways, whereas inappropriate or high-impact exercise (e.g., long-distance running and jump rope) accelerates microvascular shearing and triggers acute localized synovitis flares (68). To safely implement this, clinical consensus advocates a “step-up” strategy—initiating non-weight-bearing aerobic training to reduce visceral fat reservoirs before introducing low-load progressive resistance training (PRT) (69). Ultimately, structured PRT reverses RC by inducing myofibrillar hypertrophy, directly translating to enhanced joint stability and marked reductions in longitudinal HAQ scores (32).

Thus, in the pathophysiology of RA-Related Sarcopenia, reduced physical activity is not only a consequence of inflammation and pain but may also serve as an independent risk factor for disuse-related muscle degeneration. This suggests that clinical interventions should not only control inflammation but also actively promote appropriate activity to slow muscle deterioration.

### 5.3 Metabolic alterations and mitochondrial dysfunction

Beyond chronic inflammation and reduced activity, RA-Related Sarcopenia is accompanied by significant metabolic alterations and mitochondrial dysfunction. In RA patient muscle tissue, persistent inflammatory stimulation and increased fatty infiltration often lead to energy metabolism imbalance, manifested as disruption of glucose and lipid metabolic pathways. This not only affects muscle energy supply but may also promote intramuscular fat deposition and muscle mass decline. Furthermore, these metabolic abnormalities can exacerbate muscle resistance to insulin signaling, further disrupting protein synthesis and energy utilization efficiency.

Increasing research focuses on mitochondrial content and oxidative metabolic function in RA patient muscles. A case-control study revealed significantly reduced mitochondrial content in RA patient muscles compared to controls. Although adjusted oxidative phosphorylation capacity showed no significant differences, overall reduced mitochondrial matrix enzyme activity suggests diminished mitochondrial numbers may constitute a key component of RA muscle metabolic dysfunction (70). Concurrently, systemic metabolic and gene expression analyses confirmed significant abnormalities in energy metabolism-related genes and metabolites in RA patient muscles, indicating disrupted regulation of muscle energy metabolism in RA-Related Sarcopenia (43). These mitochondrial and metabolic dysfunctions likely arise not only from inflammation itself but also from interactions with prolonged low activity levels and fat infiltration, synergistically driving muscle functional decline.

At the sub-cellular level, this chronic energy failure is initiated by excessive mitochondrial reactive oxygen species (ROS) accumulation, which collapses the mitochondrial membrane potential and curtails ATP synthesis (71). This bioenergetic deficit subsequently triggers Caspase-3 activation, executing the specific cleavage of actomyosin complex to cause structural muscle mass loss (72). Clinically, this localized organelle distress is characterized by a distinct peripheral biomarker profile across cohorts, primarily consisting of elevated serum myostatin and growth differentiation factor 15 (GDF15) combined with a marked reduction in circulating irisin (73, 74).

Thus, in the pathophysiology of RA-Related Sarcopenia, metabolic reprogramming and mitochondrial dysfunction may serve as a critical bridge linking the inflammatory state to muscle mass decline. In-depth exploration of this field holds promise for identifying novel targets for future intervention strategies.

### 5.4 Clinical context: disease activity and treatment exposure

Sarcopenia in RA is intrinsically linked to disease-specific clinical metrics. High disease activity, quantitatively measured by DAS28 or Clinical Disease Activity Index (CDAI), strongly correlates with accelerated muscle decline. Concurrently, structural joint damage directly worsens physical function, which is closely reflected in poorer HAQ (Health Assessment Questionnaire) scores and patient-reported outcomes (PROs) (75). Regarding treatment exposure, while targeted therapies such as TNF inhibitors, IL-6 inhibitors, and JAK inhibitors (targeted synthetic disease-modifying antirheumatic drugs, tsDMARDs) can mitigate muscle catabolism by aggressively suppressing systemic inflammation, current evidence suggests their capacity to directly reverse existing sarcopenia without adjunct exercise remains limited (76). Therefore, continuous monitoring of these RA-specific indices is essential for early risk stratification.

Clinically, there is a distinct linkage between systemic inflammation and muscle deterioration. Studies have demonstrated an inverse relationship between disease activity indices and muscle health, where higher DAS28 scores are significantly associated with reduced appendicular skeletal muscle mass and impaired HGS (77). Consequently, this compounded musculoskeletal deficit translates directly to poorer functional outcomes. RA patients with concomitant sarcopenia consistently report significantly higher HAQ scores compared to non-sarcopenic RA patients, reflecting profound physical disability and a severely diminished quality of life.

## 6 Management and intervention strategies for RA-related sarcopenia

**Figure 5:**
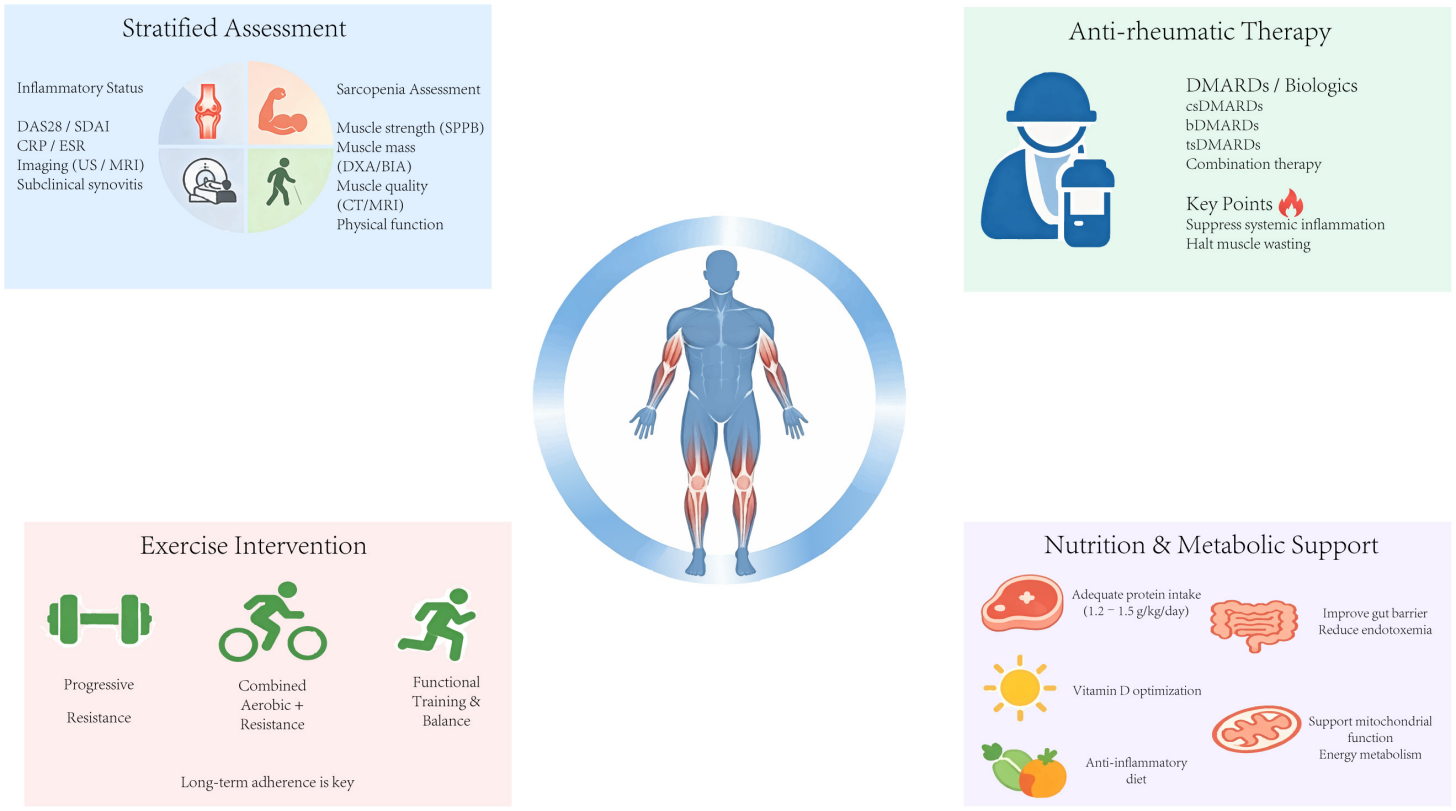
Stratified management framework for RA-related sarcopenia. This integrated paradigm begins with a stratified assessment that combines RA-specific disease activity indices with multidimensional sarcopenia measurements including DXA for muscle mass and physical performance batteries. Clinically, aggressive anti-rheumatic therapy utilizing DMARDs or JAK inhibitors serves as the pathological foundation to suppress systemic inflammation and halt active muscle wasting, while structured exercise interventions featuring PRT act as the primary non-pharmacological driver to reverse disuse atrophy. This strategy is synergistically complemented by personalized nutritional and metabolic support, which optimizes protein intake (1.2–1.5 g/kg/day) and vitamin D levels to stimulate protein synthesis and restore mitochondrial bioenergetics. Together, this synchronized multidisciplinary network shifts the clinical management of RA from isolated joint control toward the holistic preservation of body composition and physical function.

Given that RA-Related Sarcopenia is driven by multiple mechanisms including inflammation, disuse, and metabolic abnormalities, its management and intervention should adopt a comprehensive, stratified strategy rather than relying on a single therapeutic approach. As illustrated in Figure 5, the integrated management of RA-related sarcopenia includes four interconnected pillars: stratified assessment of inflammation and sarcopenic muscle function, targeted anti-rheumatic medication, standardized exercise training, and personalized nutritional-metabolic support. Anti-rheumatic therapy acts as the core to curb systemic inflammation, with exercise and nutrition as essential adjuncts to mitigate muscle wasting and restore physical capacity.

### 6.1 Impact of inflammation control and anti-rheumatic therapy

Disease-modifying antirheumatic drugs (DMARDs) and biologics play a foundational role in managing RA-related sarcopenia by suppressing systemic inflammation. International guidelines explicitly identify sustained disease activity control as a core objective for improving long-term prognosis (78). However, pooled data from meta-analyses, systematic reviews and real-world cohort studies reveals that despite the capacity of these drugs to ameliorate general physical function via suppression of disease activity, no significant direct effects on muscle mass and lean body mass measurements have been observed (3, 79, 80). Systematic reviews show that synthetic and biologic DMARDs fail to produce consistent positive changes in lean body mass or limb muscle mass (76). Quantitative meta-analyses reveal that while biologic DMARDs effectively suppress systemic inflammation, they fail to induce statistically significant increases in absolute lean body mass (3). It is important to note, however, that the current evidence base is limited by observational designs, small sample sizes, and significant heterogeneity in patients' age, sex, and treatment durations. The magnitude of benefit for muscle recovery remains unclear, and whether specific biologic classes exert distinct metabolic effects on muscle tissue requires confirmation through higher-quality, adequately powered longitudinal studies.

The above studies verify that intensive DMARD regimens—particularly when combined with low-dose glucocorticoids for transitional therapy—can safely suppress systemic TNF-α/IL-6, the key drivers of RC. From our perspective, interindividual variability in drug response directly gives rise to distinct sarcopenia phenotypes. Patients who achieve clinical remission following combination therapy with biologic or targeted synthetic DMARDs can escape the severe sarcopenia phenotype, with recovery of muscle mass and gait speed after inflammation abates. In contrast, patients with treatment-refractory disease and inadequate drug response remain in either suspected or confirmed sarcopenia phenotypes due to persistent subclinical synovitis. In response to this phenotypic divergence, we propose an individualized clinical intervention strategy: patients with poor therapeutic response should undergo early, aggressive treatment switching (e.g., rotation of JAK inhibitors or IL-6 receptor antagonists), paired with objective muscle mass screening via DXA, to prevent irreversible myofibrillar loss.

Regarding treatment exposures, a critical distinction exists between the body composition trajectories driven by biological disease-modifying antirheumatic drugs (bDMARDs) and tsDMARDs. While bDMARDs (e.g., TNF inhibitors) effectively neutralize catabolic cytokines to halt muscle wasting, they rarely promote true anabolic recovery of lean mass, often resulting in paradoxical, fat-predominant weight gain (14). Conversely, tsDMARDs exert unique pleiotropic effects on skeletal muscle protein turnover and lipid metabolism via the intracellular JAK-STAT pathway (81, 82). These mechanistic disparities suggest tsDMARDs may offer distinct advantages in counteracting RC, though robust head-to-head quantitative trials remain urgently needed to validate their comparative functional benefits.

In pharmacological management, differentiating between morphological and functional outcomes is equally critical. While bDMARDs (e.g., TNF inhibitors) effectively neutralize catabolic cytokines to halt muscle wasting and preserve muscle mass, this pharmacological preservation alone is often insufficient to fully restore muscle strength or physical functional capacity without concurrent mechanical loading (79). Conversely, tsDMARDs are showing unique pleiotropic potential. Recent experimental evidence demonstrates that JAK inhibitors can directly attenuate IL-6/JAK/STAT activation in skeletal muscle, decrease atrogenes, and restore muscle differentiation markers, thereby promoting active muscle remodeling alongside systemic immunosuppression (83).

Given the distinct regulatory effects of different anti-rheumatic agents on lean body mass and muscle function, the phenotypic impacts and inherent limitations of three major DMARD categories are conceptually summarized in Table 3. It is crucial to emphasize that these categorizations represent evolving clinical interpretations based on current observational data, rather than definitive, evidence-graded clinical recommendations. From our perspective, glucocorticoids drive muscle wasting through a dual molecular-metabolic trap: they directly transactivate myostatin and KLF15 to upregulate MuRF1-mediated proteolysis, while concurrently precipitating insulin resistance to halt muscle protein synthesis (84, 85). Given emerging evidence linking accelerated sarcopenia progression to cumulative exposure thresholds (86), we argue that rational corticosteroid use must transition from a traditional “taper-to-stop” schedule to an assertive “bridge-and-switch” paradigm. Specifically, clinicians should strictly restrict low-dose prednisone to short-term bridging intervals ( ≤ 3 months) while aggressively expanding advanced biological or targeted synthetic DMARDs to rapidly extinguish the underlying systemic inflammatory catabolism. In addition, the therapeutic impact of DMARDs across individual sarcopenia phenotypes is fundamentally asymmetrical. While classical anti-TNF-α biologics rescue muscle quantity by halting ubiquitin-mediated proteolysis, advanced JAK inhibitors excel in restoring muscle strength and performance via rapid neurological dampening of joint pain and kinesiophobia (82, 87, 88). Consequently, sarcopenia prevalence in treated RA cohorts manifests a stark epidemiological divergence between conventional synthetic DMARD monotherapy and early biologic/targeted synthetic combinations, directly reflecting the depth of subclinical synovitis clearance.

**Table 3:** Conceptual summary of the potential effects of different DMARDs on lean body mass, muscle strength and corresponding limitations in RA patients.

Drug category	Representative drugs	Effects on lean body mass	Influence on muscle strength	limitations
csDMARDs	MTX, Leflunomide (78)	Slight improvement; reduces muscle breakdown by suppressing pro-inflammatory mediators (e.g., TNF-α)	Improvement depends on disease–activity control; minimal direct anabolic effect	Only exerts an indirect protective effect; cannot reverse established sarcopenia (76)
bDMARDs	TNF inhibitors, IL-6 inhibitors (89)	Beneficial; lowers cachexia-related factors to preserve or elevate lean mass	Marked improvement; relieves pain-stiffness and boosts exercise complianc	Prevents further muscle loss alone; pharmacotherapy cannot replace resistance-exercise-induced muscle hypertrophy (3)
tsDMARDs (JAK inhibitor)	Tofacitinib, Baricitinib, Upadacitinib (90)	Promising; modulates the myostatin pathway to increase muscle mass.	Rapidly relieves inflammation, accelerating recovery of muscle explosive power	Metabolic benefits remain incomplete; dietary intervention is required to improve myosteatosis and sarcopenic obesity (1)

csDMARDs, conventional synthetic disease-modifying antirheumatic drugs; MTX, methotrexate. bDMARDs, biological disease-modifying antirheumatic drugs; IL-6, interleukin-6; RA, rheumatoid arthritis; TNF-α, tumor necrosis factor-α; tsDMARDs, traditional synthetic disease-modifying antirheumatic drugs.

The effects and limitations summarized in this table are derived from a qualitative synthesis of current literature. They represent conceptual and clinical interpretations intended to inform ongoing research, not formal, evidence-graded clinical guidelines.

Finally, we note that the clinical interaction between refractory RA and sarcopenia is fundamentally bidirectional. While refractory disease mandates aggressive target rotation among advanced b/tsDMARDs, established sarcopenia phenotypes simultaneously undermine these therapeutic efforts. Specifically, the low muscle mass phenotype blunts the clinical response rate to biological agents by altering systemic drug distribution volumes, while the impaired muscle strength phenotype exacerbates joint instability and pain, trapping refractory patients in a vicious cycle of persistent high disease activity and functional non-response.

### 6.2 Exercise intervention: from contraindication to core strategy

With deepening understanding of RA pathophysiology, exercise intervention has evolved from an early relative contraindication to a core management strategy. Current evidence indicates that structured resistance training and combined resistance-aerobic exercise demonstrate good safety and feasibility in RA patients, significantly improving muscle strength and physical function. However, their impact on muscle mass itself is often less pronounced than improvements in functional measures. Among intervention types, high-quality randomized controlled trials (RCTs) demonstrate that PRT yields robust quantitative gains in muscle strength, though absolute hypertrophic increases in muscle mass remain modest (91). While various RCTs and prospective studies advocate for combined resistance and aerobic training (92), a critical appraisal reveals significant intervention heterogeneity across studies regarding exercise modality, intensity, and duration. Furthermore, the overall magnitude of the effect on absolute muscle mass remains modest to negligible. Crucially, most trials report only short-term outcomes; robust data concerning long-term adherence rates, sustained functional benefits, and safety profiles (e.g., exacerbation of joint damage) in diverse RA subpopulations are still lacking (93). This suggests that while functional improvement is a consistent finding, the precise clinical prescription and expected effect size for RA-related sarcopenia remain to be standardized.

When evaluating non-pharmacological interventions, it is imperative to explicitly differentiate between outcomes of muscle mass, muscle strength, and physical function. PRT remains the most efficacious therapy. Quantitative data demonstrate that the benefits of PRT are distinctly disproportionate across these domains. Structured PRT yields robust, quantifiable improvements in muscle strength (with trials reporting increases in dynamic 1-repetition maximum of up to 44% following a 6-month progressive loading program) and physical function. However, the corresponding increases in absolute appendicular muscle mass are often markedly modest. This critical divergence underscores that improvements in muscle intrinsic properties and neural adaptations precede macroscopic hypertrophy, establishing PRT primarily as a strength-restoring modality (94).

### 6.3 Nutrition and metabolic support

Nutritional and metabolic support serves as a crucial adjunct in managing RA-Related Sarcopenia. A narrative review indicates that protein intake is widely recognized as beneficial for maintaining muscle protein synthesis capacity in overall sarcopenia management (95), while meta-analyses strongly support protein supplementation for geriatric sarcopenia, high-quality RCTs evaluating specific quantitative protein targets in RA cohorts are currently lacking, limiting the formulation of disease-specific guidelines (96). Specifically in RA populations, cross-sectional data show associations between low serum vitamin D levels and a higher prevalence of severe sarcopenia and impaired lower limb function (97, 98). Nevertheless, these findings remain largely speculative and associative. The causal role of vitamin D supplementation or specific protein intake targets in reversing RA-sarcopenia lacks robust confirmation from high-quality RCTs, leaving the specific size of benefit unclear (99). Similarly, while anti-inflammatory dietary patterns possess theoretical mechanistic appeal for improving metabolic environments, empirical evidence demonstrating their specific clinical efficacy and optimal dosage in RA cohorts is currently insufficient.

Furthermore, we propose that dietary elements and pre/probiotics act as metabolic and immunomodulatory “adjuvants” that synergistically lock with DMARD mechanisms to rescue muscle phenotypes. Specifically, while advanced DMARDs chemically switch off systemic cytokine cascades, prebiotic-derived short-chain fatty acids (SCFAs) concurrently provide the critical mitochondrial ATP substrate required to fuel downstream muscle protein synthesis (100). Immunologically, microbial mitigation of TLR4-mediated endotoxemia cross-attenuates peripheral catabolic drifts (101). Consequently, this microenvironment stabilization synergizes with advanced tsDMARDs/bDMARDs to accelerate the rescue of muscle quantity by arresting proteolysis, while concurrently mitigating localized joint nociception to restore muscle strength and performance.

### 6.4 Comprehensive management model for RA patients

Given the multifactorial nature of RA-Related Sarcopenia, its effective management relies on a multidisciplinary collaborative approach. Integrating disease control from rheumatology, exercise interventions from rehabilitation medicine, and personalized nutritional support facilitates a shift from solely “joint control” to a management model emphasizing both “function and body composition.” A comprehensive management model guided by functional outcomes and quality of life may represent the future direction for clinical practice in RA-Related Sarcopenia.

## 7 Challenges and future directions

Despite increasing research on RA-related sarcopenia in recent years, significant gaps remain in conceptual definition, assessment pathways, and intervention evidence, limiting the integration of findings and clinical translation.

### 7.1 Limitations in conceptual definition and assessment systems

Current RA-related sarcopenia studies pre-dominantly adopt definitions and diagnostic criteria for sarcopenia in the general population. These frameworks, primarily established based on age-related muscle degeneration, remain controversial regarding their adequacy in reflecting disease-specific muscle loss driven by chronic inflammation. Simultaneously, partial overlap in body composition and metabolic phenotypes between RC and sarcopenia leads to frequent conflation of the two conditions in research and clinical practice, further complicating conceptual definition.

At the assessment level, significant heterogeneity exists across studies regarding muscle mass measurement methods, calibration approaches, and functional indicator selection. Furthermore, RA-specific joint pain, deformities, and functional limitations may systematically interfere with muscle strength and functional test results, thereby undermining the specificity and consistency of existing diagnostic pathways in RA populations. Future research should explore assessment frameworks better suited for RA patients, emphasizing the integrated use of muscle mass and functional indicators rather than relying solely on muscle mass thresholds.

### 7.2 Shortcomings in longitudinal evidence and intervention studies

Existing research on RA-related sarcopenia remains pre-dominantly cross-sectional, lacking long-term longitudinal data to elucidate its trajectory and causal relationships with disease activity changes, treatment responses, and long-term adverse outcomes. This evidence gap limits comprehensive understanding of the clinical significance of RA sarcopenia and hinders early identification of high-risk populations.

Furthermore, the evidence base for current intervention studies remains limited. Existing research has largely focused on inflammation control or single-component exercise interventions, often involving small sample sizes. Few studies have prioritized sarcopenia or muscle function improvement as primary outcomes, and results show inconsistent findings. Future research should design longitudinal intervention studies with RA-related sarcopenia as the core endpoint to systematically evaluate the synergistic effects of multi-component strategies, including anti-inflammatory treatments, exercise training, and nutritional support.

### 7.3 Research prospects for individualized and precision management

RA patients exhibit high heterogeneity in inflammatory activity levels, body composition characteristics, and functional status, suggesting that one-size-fits-all assessment and intervention strategies may fail to meet the diverse needs of individual patients. A stratified management model based on body composition phenotypes, functional status, and disease activity characteristics holds promise for enhancing the efficiency of identifying RA-related sarcopenia and improving intervention outcomes. Advancements in quantitative imaging techniques and digital health tools open new possibilities for dynamic, personalized muscle health monitoring in precision management.

Overall, RA-Related Sarcopenia should be regarded as a distinct clinical phenotype with independent significance and potential for intervention. Future research should advance collaboratively in conceptual definition, standardization of assessment systems, and development of function-outcome-oriented intervention strategies to better improve long-term functional status and quality of life in RA patients.

## 8 Discussion

This comprehensive review systematically elucidates the multi-dimensional networks connecting RA to secondary sarcopenia, fulfilling our primary objectives to map out the pathogenic cross-talk, systemic diagnostics, and integrated therapeutic matrix. A synthesis of current clinical evidence underscores three critical thematic pillars that define the contemporary management landscape, while simultaneously highlighting prominent limitations that remain to be addressed in future clinical investigations.

First, our appraisal clarifies that the development of muscular degradation in RA is not merely a localized consequence of synovitis, but an accelerated convergence of chronological aging and disease-specific cachexia. In the aging host, canonical primary sarcopenia driven by progressive motor neuron loss, localized denervation-reinnervation failure, and a steady decline in sex hormones provides a vulnerable physiological baseline. However, when juxtaposed with RA, this age-related vulnerability is drastically compounded by a persistent “inflammaging” drift. The chronic systemic hyper-expression of TNF-, IL-6, and IL-1β shifts muscle homeostasis from a low-grade age-related catabolism into an aggressive, accelerated proteolysis driven by the NF-B-mediated ubiquitin-proteasome system. Furthermore, aging blunts the muscular regenerative potential by inducing muscle stem cell (satellite cell) senescence, an anabolic resistance that is further aggravated by prolonged physical deconditioning and disease-driven kinesiophobia. Consequently, the intersection of aging and RA-specific pathophysiology creates a synergistic “double-hit” model on skeletal tissue, meaning that elderly RA cohorts exhibit far worse muscle quality depletion and faster physical performance failure than age-matched healthy controls.

Second, a critical evaluation of current diagnostic frameworks reveals a profound mismatch between generic sarcopenia definitions and the clinical realities of the RA population. Over the past decade, criteria established by international working groups (e.g., EWGSOP2, AWGS) have rightfully shifted from purely muscle mass-centric assessments to a muscle function-first hierarchy, prioritizing HGS and physical performance. While this conceptual fluidity represents significant diagnostic progress, its application in RA introduces substantial diagnostic fractures and structural limitations. Localized joint deformities, tenosynovitis, and acute hand pain frequently compromise HGS values, causing an artifactual overestimation of severe sarcopenia that reflects mechanical joint failure rather than intrinsic muscle weakness. Conversely, the widespread use of DXA or BIA captures quantity but fails to identify muscle quality. In active RA, subclinical intramuscular lipid infiltration (myosteatosis) and localized edema can falsely mask lean mass deficits, leading to false-negative screenings. These limitations highlight an urgent diagnostic necessity: the field must transition from generic criteria toward disease-specific algorithms that decouple mechanical joint impairment from intrinsic neuromuscular sarcopenia phenotypes.

Third, this pharmacological variance highlights the indispensable role of non-pharmacological counter-measures, specifically the synergistic integration of PRT and targeted Nutritional Support. Mechanistically, PRT serves as the primary physical stimulus to reverse muscle atrophy, directly triggering mechanotransduction pathways (such as mTORC1 activation) that stimulate myofibrillar protein synthesis, upregulate muscle satellite cell proliferation, and downregulate myostatin expression. However, the hypertrophic efficacy of PRT is entirely dependent on adequate metabolic substrate availability, which is compromised in RA due to disease-driven anorexia and hypermetabolic wasting (RC). Targeted nutritional support—prioritizing high-quality protein enrichment (e.g., whey protein), branched-chain amino acids (BCAAs, especially leucine), and Vitamin D—acts as a metabolic adjuvant. Leucine directly complements PRT by chemically driving the phosphorylation of downstream translation initiation factors, while Vitamin D signaling via skeletal muscle Vitamin D receptors (VDR) optimizes calcium homeostasis, cross-amplifying the mechanical tension generated during resistance training to accelerate the rescue of muscle strength and performance.

Despite this robust physiological rationale, the clinical translation of combined PRT and nutritional support is severely hampered by structural and disease-specific limitations in RA. In patients presenting with high disease activity, active synovitis, and erosive joint destruction, the mechanical loading required for effective PRT is frequently restricted by severe joint instability and pain, trapping patients in a vicious cycle of persistent kinesiophobia and disuse muscle atrophy. Furthermore, systemic “inflammaging” can induce a state of “anabolic resistance,” wherein chronic TNF-α and IL-6 elevations blunt the capacity of skeletal muscle to respond to both exercise stimuli and hyperaminoacidemia. Consequently, overcoming refractory sarcopenia in RA demands an evolution away from isolated treatments toward a tightly synchronized, multi-modal Treat-to-Target (T2T) framework, where aggressive pharmacological synovitis clearance is structurally aligned with early, pain-titrated PRT and metabolic-nutritional profiling.

To systematically clarify the consistent effects of resistance training and nutritional support on sarcopenia reported in published studies, the core findings of three representative literatures are summarized in Table 4.

**Table 4:** Summary of therapeutic outcomes of resistance training combined with nutritional support for rheumatoid sarcopenia.

References	Study population	Intervention protocol	Key outcomes
Lichtenberg et al., (102)	Older men with osteosarcopenia	High-intensity PRT alone	Increased appendicular lean mass, HGS, leg press strength and SMI
Cochet et al., (103) (systematic review)	Elderly patients with sarcopenia	Resistance training + high-protein diet/BCAAs/vitamin D supplementation	Elevated muscle fiber cross-sectional area, SMI and grip strength; improved physical function questionnaire scores
Lander et al., (104)	Young and older adults	PRT + adequate protein intake	Alleviated age-related muscle loss and enhanced muscle anabolism

BCAAs, branched-chain amino acids; SMI, skeletal muscle mass index. HGS, hand grip strength; PRT, progressive resistance training.

Finally, our synthesis of contemporary therapeutic interventions exposes an efficacy matrix characterized by profound phenotypic asymmetry and structural disconnects. Current standard-of-care pipelines—comprising csDMARDs, b/tsDMARDs, and multi-modal lifestyle counter-measures—rarely achieve synchronized rescue across all sarcopenic phenotypes. While advanced biologic combinations (such as anti-TNF- or anti-IL-6R axes) effectively halt the catabolic drift to stabilize raw muscle quantity, they rarely prompt true anabolic recovery of lean mass and can paradoxically induce a fat-predominant weight gain that worsens sarcopenic obesity. In contrast, tsDMARDs like JAK inhibitors offer unique advantages in swiftly restoring muscle strength and physical performance by neutralizing localized nociception and kinesiophobia via neural signaling axes, yet their long-term capacity to reverse structural myofibrillar atrophy remains unvalidated.

## 9 Conclusions

In conclusion, managing rheumatoid sarcopenia requires a paradigm shift from empirical care to a synchronized, biomarker-driven strategy. Based on current clinical evidence, we deliver five decisive roadmaps for contemporary practice:

The ideal criterion: EWGSOP2 framework, integrated with hand-pain correction factors, is the most ideal for RA. Its function-first hierarchy catches early neuromuscular decay before irreversible muscle loss, while pain-titrated calibration eliminates mechanical joint artifacts that cause false-positive over-diagnostics.

Methodological reproducibility: DXA standardized against BMI (DXA-SMI/BMI) offers the highest reproducibility across shifting diagnostic criteria. This calibration normalizes fluid fluctuations caused by active rheumatoid edema, significantly outperforming BIA in preventing false-negatives.

Phenotypic biomarkers: early stratification and longitudinal monitoring rely on a specific biomarker matrix: serum myostatin and activin A track the low muscle mass phenotype; C-terminal agrin fragment (CAF) and BDNF reflect neuromuscular junction instability to monitor the impaired muscle strength phenotype; while IL-6 and hs-CRP trajectories predict overall performance decline.

Impact of targeted therapies: in conventional-refractory, hard-to-treat RA phenotypes, JAK inhibitors exert a superior clinical impact on muscle preservation compared to classic biologics. Beyond blocking myocyte proteolysis via the intracellular JAK-STAT axis, their rapid suppression of central nociception completely eliminates kinesiophobia, opening a responsive therapeutic window for physical rehabilitation.

Multi-modal integration: ultimately, maximizing functional remission demands an integrated T2T multi-modal network. In this synchronized framework, advanced pharmacological inflammation control is immediately interlocked with personalized, leucine-rich dietary strategies and early, pain-titrated PRT to permanently stabilize long-term muscle architecture.
